# Chlorogenic Acid Functions as a Novel Agonist of PPAR*γ*2 during the Differentiation of Mouse 3T3-L1 Preadipocytes

**DOI:** 10.1155/2018/8594767

**Published:** 2018-12-03

**Authors:** Shu-guang Peng, Yi-lin Pang, Qi Zhu, Jing-he Kang, Ming-xin Liu, Zheng Wang

**Affiliations:** ^1^College of Bioscience and Biotechnology, Hunan Agricultural University, 410128 Changsha, China; ^2^Research Institute of Hunan Tobacco Science, Changsha 410007, China; ^3^College of Plant Protection, Hunan Agricultural University, 410128 Changsha, China; ^4^Key Laboratory of Agro-Ecological Processes in Subtropical, Institute of Subtropical Agriculture, Chinese Academy of Sciences, Hunan, Changsha 410125, China

## Abstract

Rosiglitazone (RG) is a well-known activator of peroxisome proliferator-activated receptor-gamma (PPAR*γ*) and used to treat hyperglycemia and type 2 diabetes; however, its clinical application has been confounded by adverse side effects. Here, we assessed the roles of chlorogenic acid (CGA), a phenolic secondary metabolite found in many fruits and vegetables, on the differentiation and lipolysis of mouse 3T3-L1 preadipocytes. The results showed that CGA promoted differentiation* in vitro* according to oil red O staining and quantitative polymerase chain reaction assays. As a potential molecular mechanism, CGA downregulated mRNA levels of the adipocyte differentiation-inhibitor gene* Pref1* and upregulated those of major adipogenic transcriptional factors (*Cebpb* and* Srebp1*). Additionally, CGA upregulated the expression of the differentiation-related transcriptional factor PPAR*γ*2 at both the mRNA and protein levels. However, following CGA intervention, the accumulation of intracellular triacylglycerides following preadipocyte differentiation was significantly lower than that in the RG group. Consistent with this, our data indicated that CGA treatment significantly upregulated the expression of lipogenic pathway-related genes* Plin* and* Srebp1* during the differentiation stage, although the influence of CGA was weaker than that of RG. Notably, CGA upregulated the expression of the lipolysis-related gene* Hsl*, whereas it did not increase the expression of the lipid synthesis-related gene* Dgat1*. These results demonstrated that CGA might function as a potential PPAR*γ* agonist similar to RG; however, the impact of CGA on lipolysis in 3T3-L1 preadipocytes differed from that of RG.

## 1. Introduction

Diabetes mellitus is a chronic degenerative metabolic disease that seriously affects human health and has reached epidemic proportions over the last 30 years [1]. Concomitant with the prevalence of obesity and increased lifespan in industrial countries, the incidence of type 2 diabetes has risen rapidly worldwide [2], with the number of people suffering from diabetes globally expected to rise to >600 million within the next 25 years [3]. Currently, China has the highest number of affected individuals, of whom 90% are afflicted with type 2 diabetes mellitus [4, 5]. A primary treatment strategy for type 2 diabetes relies upon improving insulin sensitivity, such as with the thiazolidinedione-class drug rosiglitazone (RG), a powerful insulin sensitizer. As a ligand of the nuclear receptor peroxisome proliferator-activated receptor-gamma (PPAR*γ*), RG promotes the transcription of downstream genes of PPAR*γ* [2]; however, long-term use of RG reportedly causes severe cardiovascular events and increased bone-fracture rates [6, 7]. Additionally, RG causes a significant weight increase in overweight subjects with type 1 diabetes [8]. Therefore, the safety of RG has been challenged. Accordingly, although RG is still utilized to treat patients suffering from type 2 diabetes in China [9, 10], it has been withdrawn from the European market [11]. To address this issue, researchers have focused on identifying new PPAR*γ* agonists [11, 12] and insulin sensitizers as RG substitutes.

Many types of phenols have been advocated as natural remedies (*e.g*., eugenol or capsaicins) or dietary supplements (*e.g*., methoxypsoralen). The burden, metabolism, and biological effects of these dietary polyphenols are gradually gaining scientific and public attention and have been well studied, with results clarifying the margin of safety between a safe dose and the minimal dose necessary to produce significant adverse effects [13–15].

Epidemiological studies demonstrated that chlorogenic acid (CGA), a type of dietary polyphenol present in high quantities in plants and constituting the main active ingredient in many fruits, vegetables, and plants [16, 17], possesses numerous pharmacological activities, including those associated with antioxidative [18], anticancer [19–21], hypolipidemic [22], antihypertensive [23], anti-inflammatory [24], and hypoglycemic effects. Moreover, studies reported effects related to anti-insulin resistance and obesity [25], and protection against plant pathogenic fungi [26], as well as antimicrobial effects [27], inhibition of bile-duct ligation-induced liver injury [28], and attenuation of lipopolysaccharide-induced acute kidney injury [29]. Furthermore, CGA reportedly exhibits neuroprotective activity [30], and hypoxia-induced angiogenesis [31], and extends the lifespan of* Caenorhabditis elegans* [32]. Accumulating studies demonstrate that CGA exhibits antiobesity function by adjusting obesity-related adipokine levels, upregulating *β*-oxidation of fatty acids in the liver, and downregulating fatty acid and cholesterol biosynthesis in obese mice fed a high-fat diet (HFD) [16, 33]. Our previous study showed that CGA improved obesity-related metabolic disorders by upregulating* Pparg2* expression and inhibiting the nuclear factor (NF)-*κ*B-signaling pathway in the adipose tissue of obese rats induced by a HFD [17]. Therefore, we hypothesized that CGA might function as a PPAR*γ* agonist similar to RG, yet play a different regulatory role in preadipocyte differentiation to adipocytes.

Here, we used 3T3-L1 cells as an experimental model to explore the roles of CGA on lipogenesis. Specifically, we assessed the influence of CGA on 3T3-L1 cell proliferation and the expression of transcription factors [*Pparg2*,* CCAAT/enhancer binding protein beta *(*Cebpb*), and* sterol regulatory element-binding protein* (*Srebp*)1] and the key adipocyte-differentiation-related gene preadipocyte factor 1 (*Pref1*) during adipocyte differentiation. These results associated with CGA-mediated differentiation of mouse 3T3-L1 cells provide meaningful referential data for the rational use of CGA and the improvement of dietary structure.

## 2. Materials and Methods

### 2.1. Chemicals and Materials

Dulbecco's modified Eagle medium (DMEM), fetal bovine serum (FBS), streptomycin, and penicillin were acquired from Thermo Fisher Scientific (Waltham, MA, USA). CGA (*⩾*95% purity), rosiglitazone (RG; *⩾*98% purity), and GW9662 (*⩾*99% purity) were purchased from Sigma-Aldrich (St. Louis, MO, USA), dissolved in dimethyl sulfoxide formulated as a 10 mM stock solution, and stored at -20°C. The rabbit monoclonal antibody against PPAR*γ* (81B8) was purchased from Cell Signaling Technology (Danvers, MA, USA), and antibodies against *β*-actin, proliferating cell nuclear antigen (PCNA), horseradish peroxidase (HRP)-conjugated goat anti-mouse IgG, and HRP-conjugated goat anti-rabbit IgG were purchased from Proteintech (Rosemont, IL, USA).

### 2.2. Cell Culture

Undifferentiated mouse 3T3-L1 preadipocytes were purchased from the Cell Resource Center, Shanghai Institutes for Biological Sciences, Chinese Academy of Sciences (Shanghai, China), and cells were passaged 17 times. 3T3-L1 cells were cultured in high-glucose DMEM containing 10% FBS and 1% penicillin/streptomycin and incubated at 37°C in an atmosphere with 5% CO_2_.

### 2.3. Cell-Viability Analysis

3T3-L1 cells were seeded into 96-well plates at a density of 5 × 10^3^ cells/well and incubated overnight. Cells were then incubated with different concentrations of CGA, and after 24 h, 48 h, and 72 h, cell viabilities were determined by counting living and dead cells using the trypan blue dye (0.05% solution)-exclusion method analyzed with a Bright-Line Hemacytometer (Sigma-Aldrich, USA) and cell counting kit (CCK)-8 (Dojindo, Kumamoto, Japan), respectively. After a 4 h incubation with 10 *μ*L CCK-8 reagent, cell viability was calculated by measuring the optical density at 450 nm (Varioskan Flash; Thermo Fisher Scientific).

### 2.4. Induced Differentiation of Mouse 3T3-L1 Preadipocytes

3T3-L1 cells were seeded into a 6-well plate at a concentration of 2 × 10^5^ cells/well. Upon reaching ~90% confluence, the medium was replaced with fresh high-glucose DMEM containing 10% FBS and 1% penicillin/streptomycin, followed by incubation at 37°C in 5% CO_2_ for 2 days and culture in cell-differentiation medium 1 (fresh high-glucose DMEM containing 10% FBS, 0.5 mM 3-isobutyl-1-methylxanthine, 1 *μ*M dexamethasone, and 5 *μ*g/mL insulin) containing 20 *μ*M CGA, RG (positive control group), or GW9662 (negative control group). The differentiation time was calculated from the day at which the medium was changed. After 2 days, the medium was exchanged with cell-differentiation medium 2 (fresh high-glucose DMEM containing 10% FBS and 5 *μ*g/mL insulin) containing the same combination of chemicals described. After another 2 days, cell-differentiation medium 2 was removed and replaced with cell-differentiation maintenance medium (CDMM) containing fresh high-glucose DMEM and 10% FBS. CDMM was changed every 2 days until the majority of the preadipocytes had differentiated into adipocytes and obvious lipid droplets were observed in the mature fat cells.

### 2.5. Oil Red O (ORO) Staining

The lipid accumulation in differentiated 3T3-L1 cells was observed by ORO staining on day (D)4, D6, D8, and D10 of differentiation. Cells were fixed with 4% formaldehyde for 30 min to 60 min after two washes with phosphate-buffered saline (PBS) and stained in ORO solution for 1 h after another two washes, followed by several rinses with 75% alcohol to remove excess dye. The cells were then washed several times with ultrapure water, and photomicrographs were acquired at 200× magnification using a system incorporated in the DMI3000B inverted microscope (Leica, Wetzlar, Germany).

### 2.6. Triglyceride Assay

Intracellular triglycerides were evaluated using a triglyceride assay kit (GPO-POD; Applygen Technologies, Beijing, China) according to manufacturer protocol.

### 2.7. RNA Preparation and Quantitative Real-Time Polymerase Chain Reaction (qPCR)

Total RNA was isolated from test cells using TRIzol reagent (Thermo Fisher Scientific), with 1 *μ*g of each sample RNA used to generate cDNA using a reverse transcription reagent kit with gDNA Eraser (TaKaRa, Dalian, China) as a template for qPCR. Reactions were performed on a Step-One plus qPCR system (Applied Biosystems, Foster City, CA, USA) using SYBR Green qPCR master mix (TaKaRa). The sample was predenatured at 95°C for 30 s, followed by 40 cycles of denaturation at 95°C for 5 s and annealing at 60°C for 30 s. PCR efficiency for the primers ranged from 90% to 110%, and threshold cycle numbers (CT) were recorded for each reaction and normalized against that of* β-actin*. The primers were designed using Primer 5 software (Premier Biosoft, Palo Alto, CA, USA) and synthesized by Sangon Biotech Co., Ltd. (Shanghai, China) (Table 1).

### 2.8. Western Blot Analysis

On D0, D2, D4, D6, and D8 of 3T3-L1 cell differentiation, cells were lysed in radio immunoprecipitation assay lysis buffer (Applygen Technologies) supplemented with protease and phosphatase inhibitors on ice for 10 min after ice-cold PBS washes, followed by centrifugation at 12,000* g* for 15 min at 4°C and supernatant collection. The nuclear fraction was extracted using a Nuc-Cyto-Mem preparation kit (Applygen Technologies), and protein concentrations were assayed using a BCA assay kit (Beyotime, Beijing, China). Each sample (50 *μ*g protein) was separated by 10% gel electrophoresis before electrophoretic transfer onto polyvinylidene fluoride membranes (Bio-Rad, Hercules, CA, USA). Blots were blocked at room temperature for 2 h in blocking buffer [5% slim milk in Tris-buffered saline containing Tween20 (TBST)] and incubated with primary antibodies specific to PPAR*γ*2 (1:1000), PCNA (1:5000), and *β*-actin (1:4000) overnight at 4°C. After three washes with TBST, the membrane was incubated with the secondary antibodies [HRP-conjugated anti-rabbit (1:6000) and anti-mouse (1:4000) IgG] at room temperature for 2 h with gentle agitation. Immunoreactive bands were visualized using an enhanced chemiluminescence reagent according to manufacturer instructions (Thermo Fisher Scientific). The optical density was quantified using Quantity One software (Bio-Rad).

### 2.9. Confocal Microscopy Analysis

On D0, D2, and D8 of 3T3-L1 cell differentiation, cells were rinsed with PBS twice for 5 min and fixed with 4% paraformaldehyde for 10 min, followed by three washes with PBS and permeabilization at room temperature for 30 min with 0.5% Triton X-100. Cells were then rinsed in PBS with Tween20 three times, followed by a blocking step with PBS containing 5% FBS at room temperature for 1 h. Samples were then incubated overnight at 4°C with a 1:100 dilution of anti-PPAR*γ*2, followed by incubation with a secondary BODIPY conjugated goat anti-rabbit antibody (Cell Signaling Technology) at room temperature for 1.5 h. After incubation, cells were rinsed and stained with 4′,6-diamidino-2-phenylindole (DAPI) for 6 min, followed by a series of 5-minute washes. The cells were sealed with an antifluorescence quencher; confocal images were captured using an LSM 7DUO confocal microscope (Carl Zeiss AG, Oberkochen, Germany) and processed using ZEN Lite software (Carl Zeiss AG).

### 2.10. Statistical Analysis

All values were expressed as the means ± standard error of the mean and analyzed with the SPSS package (v16.0; SPSS, Inc., Chicago, IL, USA). One-way analysis of variance, followed by least-significant difference tests, was used to evaluate significant differences between groups, with a* P *< 0.05 considered significant.

## 3. Results

### 3.1. Effect of CGA on Mouse 3T3-L1 Preadipocyte Proliferation

**Figure 1:**
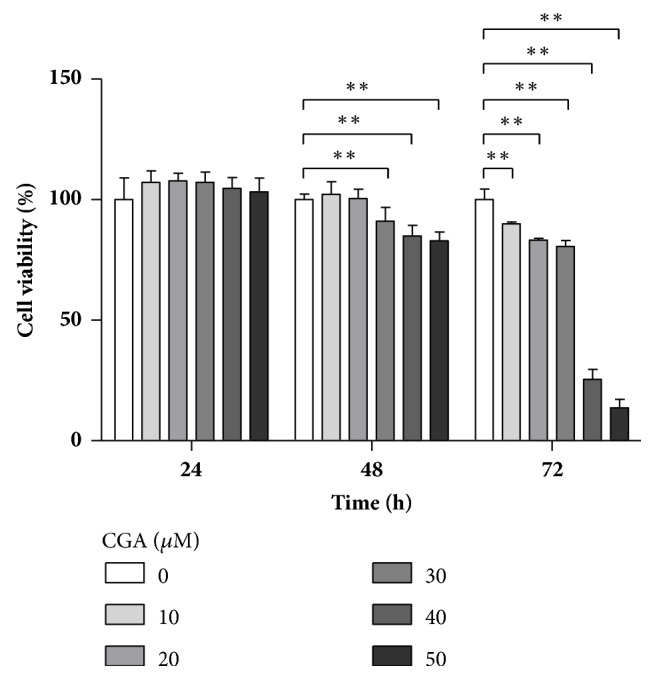
Chlorogenic acid (CGA) suppresses cell proliferation of mouse 3T3-L1 preadipocytes. 3T3-L1 preadipocytes were treated with control, 10, 20, 30, 40, and 50 *μ*M CGA for 24, 48, and 72 h at 37°C, respectively. Experiments were performed in triplicate. Data are shown as the means ± SD (n = 3). ^∗∗^*P* < 0.01.

Cell-counting results showed that CGA influenced 3T3-L1 cell proliferation, with concentrations <50 *μ*M resulting in no significant changes following incubation for 24 h and 48 h. However, increases in the incubation time to 72 h and CGA concentration *⩾*50 *μ*M resulted in significant inhibition of proliferation and a decrease in cell viability to 13.7% at 50 *μ*M CGA (Figure 1). These results indicated that CGA displayed dose- and time-dependent effects on cell viability.

### 3.2. CGA Treatment Promotes Differentiation of 3T3-L1 Preadipocytes

**Figure 2:**
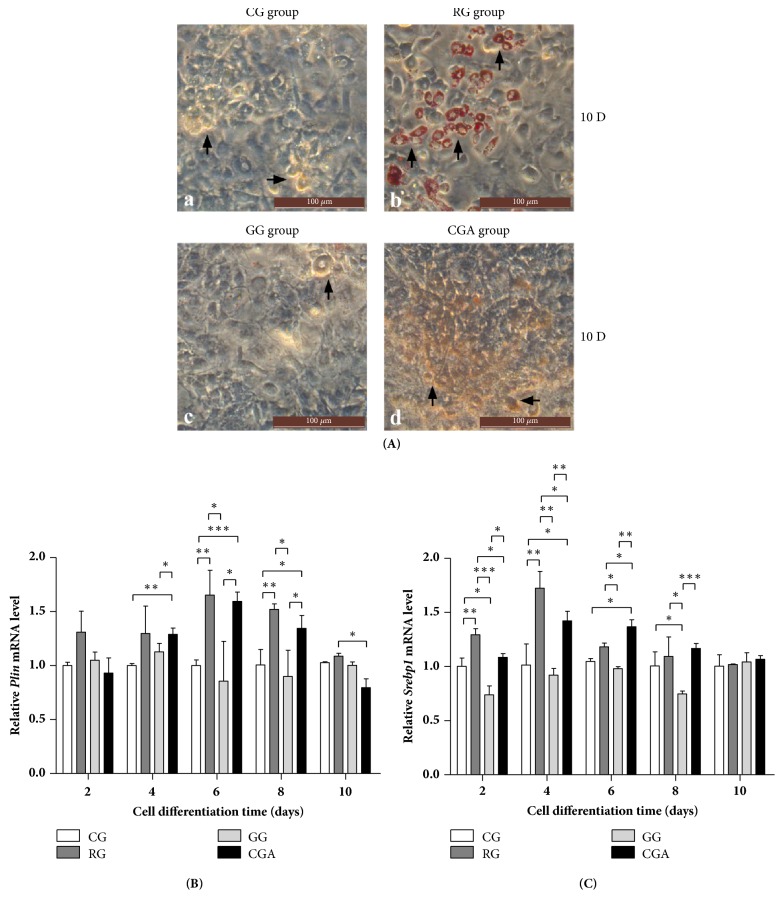
Comparative effect of chlorogenic acid (CGA) and rosiglitazone (RG) on the differentiation of mouse 3T3-L1 preadipocytes. (A) 3T3-L1 preadipocytes were cultured with or without CGA (20 *μ*M) for 10 days; then cells were stained with ORO. RG was used as a positive control. GW9662 was used as a negative control group (GG). All images are shown at 200 × magnification. (a) Control group (CG); (b) RG group; (c) GG group; (d) CGA group; the arrows in a-d indicate the differentiation of preadipocytes; (B-C) effects of CGA on the expression of lipogenic pathway-related genes during the differentiation process of mouse 3T3-L1 preadipocytes. (B)* Plin*; (C)* Srebp1*. Data are shown as the means ± SD (n = 3). ^∗^*P* < 0.05, ^∗∗^*P* < 0.01, ^∗∗∗^*P* < 0.001.

CGA was tested to investigate its ability to promote 3T3-L1 preadipocyte differentiation. We observed the accumulation of lipid droplets in differentiated cells by microscopy on D10 of differentiation, after which morphological changes were detected in RG-treated 3T3-L1 cells, which changed from a predifferentiation spindle-like shape to a round shape following differentiation [Figure 2(A)(a, b)]. Additionally, microscopy analysis of ORO-stained lipid droplets within the cells revealed obvious lipid accumulation in RG- and CGA-treated cells relative to that observed in the control and GW9662-treated groups, although CGA treatment appeared less effective than RG treatment (Figure 2). Moreover, CGA-induced adipocyte morphology differed from that of the RG group (Figure 2).

To further evaluate the effect of CGA on 3T3-L1 preadipocyte differentiation, we determined the expression of genes involved in the protection of lipid droplets from lipolysis (*perilipin*;* Plin*) [34] and* de novo *lipogenesis (*Srebp1*) [35]. Compared with the positive and negative control (GW9662) groups, following CGA intervention,* Plin* and* Srebp1* mRNA levels were significantly upregulated during the differentiation process (Figures 2(B) and 2(C)); however, consistent with ORO-staining results, CGA treatment appeared less effective than RG treatment.

### 3.3. CGA Treatment Did Not Lead to Accumulation of Excess Intracellular Triacylglyceride (TAG)

**Figure 3:**
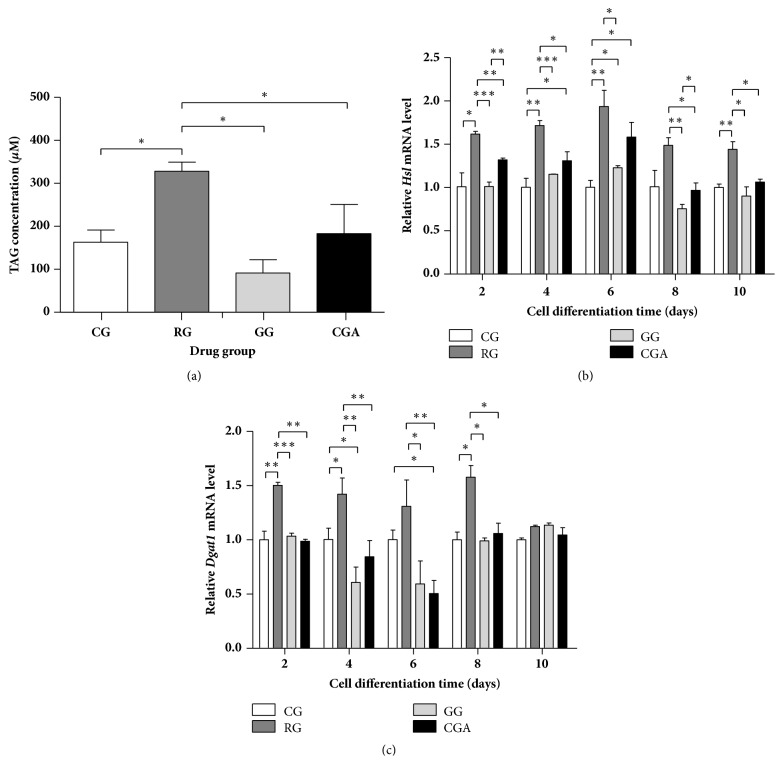
Chlorogenic acid (CGA) reduced triacylglyceride (TAG) accumulation in differentiated 3T3-L1 cells compared with the rosiglitazone group (RG). (a) Mouse 3T3-L1 preadipocytes were cultured without (control group, CG) or with CGA (20 *μ*M) for 10 days; then the cellular TAG contents were measured using a TAG determination kit. (b-c) Effects of CGA on expression of the lipolysis-related gene* Hsl* (b) and triacylglycerol synthesis-related gene* Dgat1* (c) during the differentiation process of mouse 3T3-L1 preadipocytes. RG was used as a positive control. GW9662 was used as a negative control (GG). CG, control group. Data are shown as the means ± SD (n = 3). ^∗^*P* < 0.05, ^∗∗^*P* < 0.01, ^∗∗∗^*P* < 0.001.

We determined TAG content to verify the influence of CGA on 3T3-L1 differentiation after D10. As shown in Figure 3, TAG content was significantly increased (about 2-fold) in 3T3-L1 cells treated with RG relative to that observed in the control group, whereas TAG content in CGA-treated 3T3-L1 cells was significantly decreased relative to that in the RG group (*P* < 0.05), although not significantly different from that in the control group. Additionally, treatment of differentiated cells with GW9662 (20 *μ*M) decreased triglyceride levels by 42.9% as compared with the control group. These results were consistent with ORO-staining results, in that CGA treatment appeared less effective than RG treatment. These findings indicated that CGA effectively promoted adipocyte differentiation of 3T3-L1 cells, although the accumulation of intracellular TAG following preadipocyte differentiation was significantly lower than that in the RG-treated group.

To determine the mechanistic differences between CGA- and RG-induced 3T3-L1 differentiation, we analyzed the mRNA levels of genes involved in lipolysis (*hormone-sensitive lipase*;* Hsl*) and triglyceride biosynthesis (*diacylglycerol O-acyltransferase 1*;* Dgat1*). CGA treatment significantly increased* Hsl* expression by 30.9% to 58.1% during the prophase differentiation of 3T3-L1 cells (Figures 3(b) and 3(c)), whereas treatment with 20 *μ*M CGA did not significantly alter* Dgat1* mRNA levels during the entire differentiation process relative to those observed in control and GW9662 groups. By contrast, RG treatment upregulated* Hsl* levels by 44.0% to 93.7% and* Dgat1* levels by 12.3% to 57.7% relative to those in control group. These results suggested that the reason CGA did not promote TAG accumulation might have been its upregulation of* Hsl* (lipolysis) and lack of effect on* Dgat1* (lipid synthesis) expression. These findings implied that the influence of CGA on preadipocytes differed from that of RG.

### 3.4. CGA Treatment Activates Key Genes Involved in Adipocyte Differentiation and the Transcription Factor PPAR*γ*2

**Figure 4:**
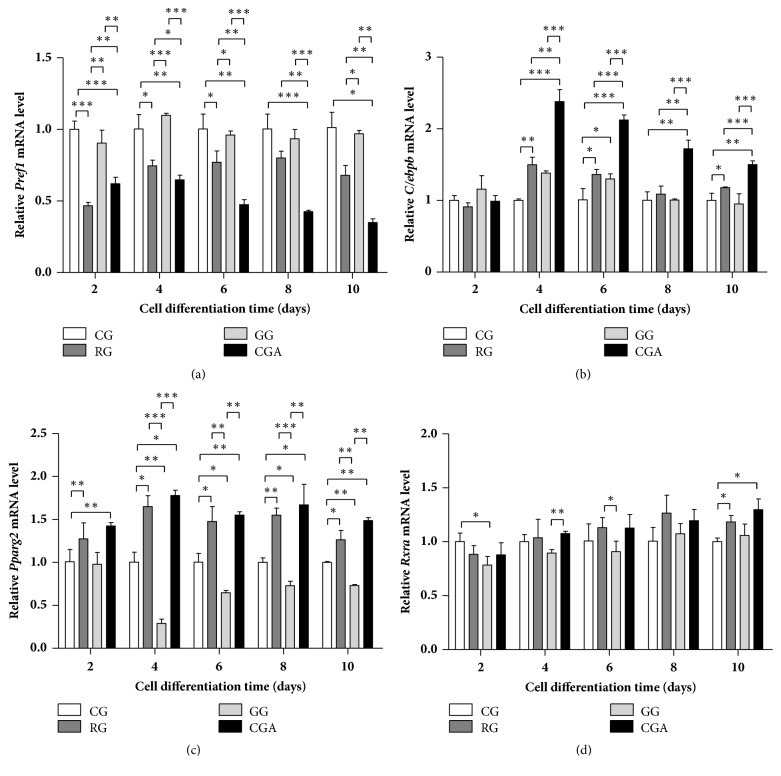
Effects of chlorogenic acid (CGA) on the expression of key differentiation-related genes during the differentiation process of mouse 3T3-L1 preadipocytes. Rosiglitazone group (RG) was used as a positive control. GW9662 was used as a negative control (GG). CG, control group. (a)* Pref1*; (b)* Cebpb*; (c)* Pparg2*; (d)* Rxra*. Data are shown as the means ± SD (n = 3). ^∗^*P* < 0.05, ^∗∗^*P* < 0.01.

To quantify the role of CGA in the expression of key adipogenesis-related genes [*Pref1* and retinoid X receptor alpha (*Rxra*)] and major adipogenic transcription factors (*Pparg2* and* Cebpb*) during adipogenesis, preadipocytes were treated with 20 *μ*M CGA during adipocyte differentiation on D2 to D10, and gene expression was analyzed by qPCR. Following CGA treatment,* Pparg2 *and* Cebpb* mRNA levels increased relative to levels in control or GW9662-treated groups, whereas* Pref1* mRNA levels decreased during the differentiation process. However, no significant change in* Rxra *mRNA levels was observed between the CGA-treated and control groups. Also, it is worth noting that the effect of CGA on* Pref1*,* Cebpb, *and* Pparg2 *mRNA levels appeared higher than that observed in the RG-treated group (Figure 4), suggesting that CGA treatment promoted 3T3-L1 cell differentiation to a level similar to that of RG treatment.

### 3.5. CGA Upregulates PPAR*γ*2 Protein Levels during Adipocyte Differentiation

**Figure 5:**
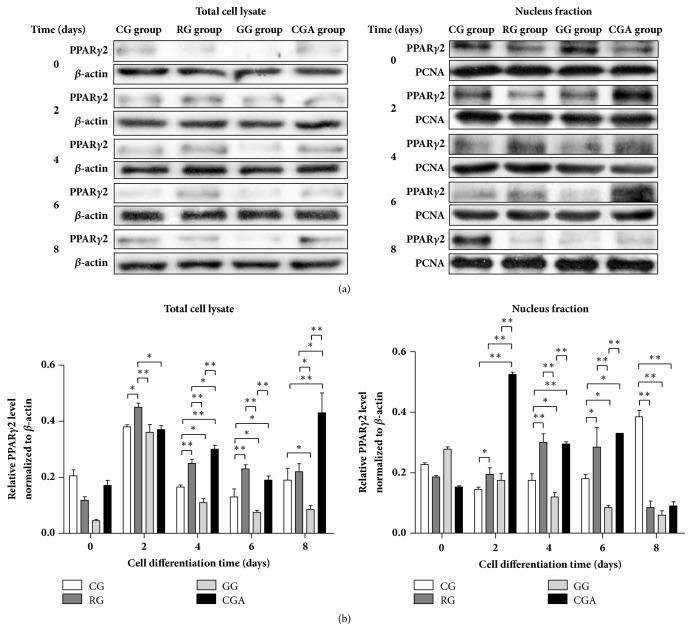
Chlorogenic acid (CGA) activates PPAR*γ*2 during the differentiation process of mouse 3T3-L1 preadipocytes. (a) Mouse 3T3-L1 preadipocytes were treated with control (control group; CG), rosiglitazone (RG), GW9662 (GG), and CGA for 2, 4, 6, and 8 days, respectively. The samples were lysed and subjected to western blot analysis with indicated antibodies. Left: total cell lysate; Right: nucleus fraction. (b) The intensity of the band was quantified using densitometric imaging. Data are shown as the means ± SD (n = 3). ^∗^*P* < 0.05, ^∗∗^*P* < 0.01.

Western blot results showed that PPAR*γ*2 levels increased significantly (by 18.42-76.92%) from D2 to D6 of differentiation following RG treatment as compared with levels in the control group (Figure 5). By contrast, CGA treatment resulted in significant increases in PPAR*γ*2 levels in 3T3-L1 cells from D4 relative to those observed in control and GW9662-treated groups. However, on D8, the difference in these levels between the RG-treated and control groups was minimal, whereas those in the CGA group were >90% higher relative to PPAR*γ*2 levels in the control (*P < *0.01), GW9662-treated (*P < *0.01), and RG-treated groups (*P < *0.05), respectively.

In the nucleus of 3T3-L1 cells on D2 to D6, PPAR*γ*2 levels in the CGA- and RG-treated groups increased relative to levels in the control and GW9662-treated groups. In particular, the highest PPAR*γ*2 levels in the CGA-treated group appeared on D2 and were >2-fold higher than those in other treatment groups (*P < *0.01). However, on D8, PPAR*γ*2 levels in both the RG- and CGA-treated groups were significantly lower (~70%) than that in the control group (*P < *0.01) and similar to that in the GW9662-treated group (*P > *0.05). These results suggested that CGA treatment promoted adipocyte differentiation through upregulated PPAR*γ*2 mRNA and protein levels.

### 3.6. CGA Affects the Subcellular Distribution of PPAR*γ*2 during Adipocyte Differentiation

**Figure 6:**
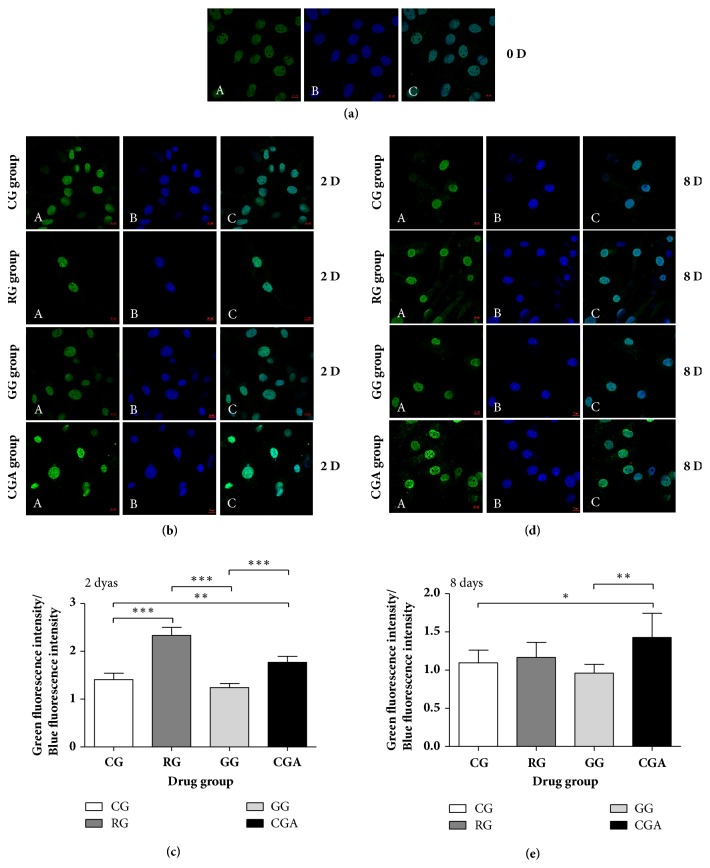
Chlorogenic acid (CGA) enhances the expression of PPAR*γ*2 during the differentiation process of mouse 3T3-L1 preadipocytes. Mouse 3T3-L1 preadipocytes were immunostained for PPAR*γ*2 protein (*green*) (A), while nuclei were simultaneously revealed by DAPI staining (*blue*) (B). Colocalization is rendered in the merge panels (*cyan*) (C). Subcellular distribution of PPAR*γ*2 and nuclei was analyzed by microscopic confocal analysis. (a) 0 days (630 ×); (b) 2 days (630 ×); (d) 8 days (630 ×). ((c) and (e)) The intensity of the green and blue fluorescence was quantified using ZEN 2 lite software. Data are shown as the means ± SD (n = 3). ^∗^*P* < 0.05, ^∗∗^*P* < 0.01, ^∗∗∗^*P* < 0.001.

PPAR*γ*2 is found specifically in adipose tissue [36]; however, to evaluate whether CGA affects PPAR*γ*2 subcellular distribution, we investigated the distribution of endogenous PPAR*γ*2 by immunostaining and confocal microscopy of mouse preadipocytes (Figure 6). On D0 and D2, immunostaining revealed PPAR*γ*2 in the nucleus, with RG treatment significantly increasing the green (PPAR*γ*2):blue (nucleus) fluorescence ratio by 65.55% as compared with that of the control group (*P < *0.01), whereas no difference was detected between the GW9662-treated and control groups (Figure 6(c)). By contrast, this ratio in the CGA-treated group was significantly (26.71%) higher than that of the control group (*P < *0.01) and significantly lower (24.07%) than that of the RG-treated group (*P *< 0.01) (Figure 6(c)). Figure 6(d) shows a robust enhancement in blue fluorescence intensity on D8 relative to that on D2, suggesting an increased PPAR*γ*2 distribution outside of the nucleus. Although we found no significant difference between the RG-treated and control groups, PPAR*γ*2 levels in the CGA-treated group were slightly higher than those in the other three groups, with green:blue fluorescence ratio 30.17% higher than that of the control group (*P *< 0.05), 48.6% higher than that of the GW9662-treated group (*P *< 0.01), and 22.47% higher than that of the RG-treated group (*P *> 0.05) (Figure 6(e)). These results indicated that CGA and RG promoted PPAR*γ*2 expression and subcellular distribution similarly during adipocyte differentiation.

## 4. Discussion

RG is a full agonist of PPAR*γ*, activates the PPAR*γ* nuclear receptor, and is widely used as a therapeutic agent for type 2 diabetes. However, studies indicate that partial PPAR*γ* agonists have lower risks of causing side effects (*e.g*., edema, fractures, and heart failure) relative to full PPAR*γ* agonists, although they exhibit similar effects associated with insulin sensitivity. Therefore, it is necessary to identify safer and more effective partial PPAR*γ* agonists [37, 38].

Many studies report that the CGA complex can be promoted as an active ingredient in the nutritional management of obesity [16, 39, 40]. In the present study, we investigated whether CGA could also affect preadipocyte differentiation through its lipid-lowering effect, as well as the molecular mechanism associated with CGA-mediated adipogenesis. We treated mouse 3T3-L1 preadipocytes with different doses of CGA, significant inhibition of 3T3-L1 cell viability in a time- and dose-dependent manner (Figure 1).

Lee* et al. *[41] reported changes in morphology of 3T3-L1 preadipocytes from a predifferentiation spindle-like shape to a round shape during early differentiate, with subsequent development of lipid droplets indicating preadipocyte differentiation into adipocytes and their accumulation signaling adipocyte maturity [42]. In the present study, ORO staining indicated that CGA promoted adipocyte differentiation, albeit to a lesser degree than that observed following RG treatment [Figure 2(A)(a)]. We found that the shape of the lipid droplets differed in the CGA-treated group relative to those in the RG-treated group (*i.e*., small and not ring-like) [Figure 2(A)(a, d)] and implying that lipid accumulation in the CGA-treated group differed from that in the RG-treated group. Moreover, this morphological change was consistent with those reported previously [40].

According to the biological processes associated with adipocyte differentiation, we evaluated the expression levels of* Plin* and* Srebp*, which comprise important factors related to lipid homeostasis in 3T3-L1 cells. Lipid droplets store a large amount of TAGs, thereby regulating the storage and hydrolysis of lipids in mammalian adipocytes. The stability of lipid droplets is dependent upon PLINs, the most well-known coat proteins embedded in the phospholipid monolayer of lipid droplets [43] and that bind to and stabilize newly formed lipid droplets in order to protect them from breakdown by HSL [34, 43]. Ruiz* et al*. [38] reported that SREBP1 levels are significantly elevated in obese patients and animal models of obesity and type 2 diabetes, with other studies indicating that SREBP1 contributes to hepatic lipid accumulation and insulin resistance. In the present study, we found that CGA treatment significantly enhanced* Plin* and* Srebp1* expression as compared with levels in the control group, thereby contributing to lipid accumulation in 3T3-L1 cells. However, levels of both genes following CGA treatment were significantly lower than those in the RG-treated group (Figures 2(B) and 2(C)), suggesting increased lipid accumulation following RG treatment relative to that observed after CGA treatment.

Interestingly, Zheng* et al*. [39] demonstrated that CGA treatment decreased serum levels of TAG, a marker for lipid homeostasis, in mice, and that hepatic TAG concentrations were decreased by CGA + caffeine administration. Moreover, a previous study reported that CGA treatment decreased TAG levels in the liver and plasma of Sprague-Dawley rats on a high-energy diet [44]. In the present study, the TAG content of CGA-treated 3T3-L1 cells increased by only 12.25%, whereas TAG levels increased significantly by 101.02% following RG treatment relative to levels in the control group (Figure 3(a)). These results suggested that CGA treatment promoted adipocyte differentiation but had a weak effect on lipid accumulation in 3T3-L1 cells.

The* Hsl* gene is expressed in adipose tissue, where it hydrolyzes stored triglycerides to free fatty acids and mobilizes the stored lipids [45]. Therefore, HSL constitutes the rate-limiting enzyme in TAG catabolism, whereas DGAT1 is the key enzyme associated with TAG synthesis [17]. Figure 2(B) shows similar* Plin* expression between the CGA- and RG-treated groups during the differentiation process, whereas* Hsl* expression was lower in the CGA-treated group relative to that in the RG-treated group (Figure 3(b)). These results suggested that the rate of lipid degradation following CGA treatment was lower than that following RG treatment, contradicting the results of ORO and TAG staining (Figures 2(A) and 3(a)). However, it is worth noting that* Dgat1* expression in the RG-treated group was ~1.5-fold higher than that in the CGA-treated group from the initial stage of differentiation to D8 and that* Dgat1* expression in the CGA-treated group was similar to that in the control group. On D6 of differentiation,* Dgat1* expression in the CGA-treated group was lower than that in the GW9662 group (Figure 3(c)), implying that lipid synthesis following CGA treatment was inhibited as compared with that following RG treatment. Additionally, during the primary differentiation stage,* Srebp1* expression in the CGA-treated group was lower than that in the RG-treated group (Figure 3(c)). This might represent one of the mechanisms associated with the differences in the results of ORO staining observed between the CGA- and RG-treated groups and suggests that CGA intervention led to the accumulation of intracellular TAG during adipocyte differentiation, although to levels significantly lower than those in the RG-treated group.

Moreover, we observed that expression of key adipocyte-differentiation-related factors (*Pref1*,* Pparg2*, and* Cebpb*) changed during adipocyte differentiation. Our results agreed with a previous study reporting abundant* Pref1* expression in preadipocytes but a dramatic decrease in these levels during adipocyte differentiation [46]. Moreover, constitutive expression of* Pref1* in preadipocytes inhibits adipocyte differentiation [46]. PREF1 obstructs adipocyte differentiation by combining the promoter regions of* Cebpb* and* Cebpd* and inactivating C/EBP*α* and PPAR*γ* [47]. Furthermore,* Pref1 *overexpression decreases adipocyte-marker expression [46], and dexamethasone-mediated inhibition of* Pref1* expression in preadipocytes activates PPAR*γ* and stimulates preadipocyte differentiation and maturation [48]. As an important transcription factor and master regulator of adipogenesis, PPAR*γ* plays an important role in regulating the expression of genes involved in fatty acid *β* oxidation, lipid homeostasis, and controlling adipogenesis [49].

PPAR*γ* has at least two subtypes: PPAR*γ*1 is expressed in most tissues, and PPAR*γ*2 is expressed specifically in adipose tissue [36]. C/EBP*α* shares a pathway with PPAR*γ* associated with regulating preadipocyte differentiation, with C/EBP*α* activity dependent upon PPAR*γ* status. Both proteins regulate the expression of* activating protein (Ap)-2*,* Fas*, and* Hsl*, as well as other factors related to preadipocyte differentiation [50]. Additionally, PPAR*γ* promotes the transformation of cultured myoblasts into adipocytes, especially when coexpressed with C/EBP*α* [41]. During the early stage of adipocyte differentiation,* Cebpb* is highly expressed and initiates mitotic clonal expansion to enable entry of preadipose cells into the cell-proliferation cycle. After approximately two rounds of mitosis, cells exit this cycle and enter the differentiation stage, at which point C/EBP*β* activates the expression of* Pparg* and other adipokines by binding to their promoter regions [51–54]. Additionally, C/EBP*β* can also promote non-fat cell differentiation into adipocytes [55]; however, this requires PPAR*γ* formation of a dimer with RXR*α*, which promotes transcriptional activity via binding to DNA [56]. PPAR*γ*1 and PPAR*γ*2 effectively stimulate adipocyte differentiation, although, under low-ligand (RXR*α*) concentrations, PPAR*γ*2 stimulation of adipose-tissue formation is significantly stronger than that of PPAR*γ*1 [57]. Moreover, Ren* et al*. [58] demonstrated that PPAR*γ*2 rather than PPAR*γ*1 plays a role in promoting cell differentiation.

In the present study, CGA and RG treatment, respectively, downregulated* Pref1* expression and upregulated* Pparg2* and* Cebpb* expression in all stages of 3T3-L1 cell differentiation and relative to levels observed in the control and GW9662-treated groups (Figures 4(a)–4(c)), whereas only minimal increases in* Rxra *levels were observed relative to controls (Figure 4(d)). These expression levels during adipocyte differentiation were consistent with those reported previously [46, 49, 51–54]. Notably, the effect of CGA treatment was more pronounced than that of RG treatment, with CGA treatment having a greater effect on* Pref1* expression. These data suggested that the number of preadipocytes in the CGA- and RG-treated groups was lower than that in the two control groups and that the majority of these adipocytes were able to differentiate into mature adipocytes. This finding was consistent with the results of ORO staining (Figure 2(A)). Additionally, several studies reported that downregulating* Plin*,* Srebp*,* Pparg*, and* Cebpa* inhibits adipogenesis in 3T3-L1 preadipocytes [34, 54, 59–61]. The results of the present study indicated that CGA treatment promoted adipogenesis specifically by affecting activation of adipogenic transcription factors and regulating adipogenesis-related gene expression.

Besten* et al*. [62] reported that short-chain fatty acids (SCFAs) prevent and reverse HFD-induced obesity in mice via a PPAR*γ*-dependent switch from lipid synthesis to fat oxidation. SCFAs also stimulate mitochondrial fatty acid oxidation by activating the uncoupling protein 2 (UCP2)-AMPK-acetyl-CoA carboxylase (ACC) pathway in 3T3-L1 preadipocytes. Moreover, activating or inhibiting PPAR*γ* activity via the PPAR*γ* agonist RG or the PPAR*γ* antagonist GW9662 abolishes SCFA-induced increases in UCP2-pAMPK-pACC signaling [63]. In the present study, we observed significantly lower TAG accumulation in 3T3-L1 cells following CGA treatment relative to that following RG treatment; however, whether this was due to CGA-mediated activation of UCP2-pAMPK-pACC signaling to promote *β* oxidation of fatty acids remains unknown.

Analysis of PPAR*γ*2 levels revealed significant increases in* Pp*a*rg*2 expression in both CGA- and RG-treated groups, and that in the GW9662-treated group was significantly lower, relative to that observed in the control during the course of adipocyte differentiation and especially on D4 (Figure 4(c)). Our analysis of PPAR*γ*2 levels agreed with qPCR results (Figures 4(c), 5, and 6) and with those reported previously [17]. These findings supported a role for CGA in promoting adipocyte through PPAR*γ*2 expression and activity.

Zheng* et al*. [39] reported that supplementation of culture medium with 0.2% CGA reduced hepatic* Pparg* expression in mice, and Ryohei* et al*. [64] demonstrated that 5% caffeine downregulated PPAR*γ* levels; however, the major bioactive constituent of CGA in coffee extract showed no effect on* Pparg *expression at concentrations of 100 *μ*M. These results were inconsistent with the findings reported in the present study. Notably, numerous studies reported results consistent with our findings [17, 22, 25]. Sanchez* et al.* [22] reported that CGA acted as either an insulin secretagogue or a PPAR*α*/*γ* dual agonist, and Ghadieh* et al.* [25] demonstrated that CGA/chromium supplementation alleviates insulin resistance. Additionally, our previous* in vivo *study [14] showed that supplementation with either low or high doses of CGA (20 and 90 mg/kg, respectively) significantly increased adipose-tissue PPAR*γ*2 mRNA and protein levels, with these levels of CGA supplementation equivalent to physiological concentrations present in humans [17]. Moreover, Du* et al. *[65] demonstrated that intravenous injection of a 5-fold higher level of CGA than the recommended daily amount induced inflammation reactions and oxidative-stress injury in rats, with these findings supporting our use of 20 *μ*M CGA in the present study. These findings suggest that the CGA concentration used in the present study was significantly lower than those of previous studies. Furthermore, the majority of CGA is hydrolyzed to caffeic acid and quinic acid before being absorbed in the gastrointestinal tract by gut microbiota esterases in both the small and large intestine [63]. Therefore, differences in dosage, CGA hydrolysis, or differentiation conditions might have contributed to inconsistencies in results. Nevertheless, our findings showed that CGA treatment activated PPAR*γ* to a degree similar to RG treatment, potentially explaining the ability of CGA to increase insulin sensitivity and inhibit chronic inflammation caused by obesity [17, 21, 22, 25].

Previous studies reported strong links between PPAR*γ*2 and the inflammatory response associated with obesity. PPAR*γ*2 inhibits the inflammatory response via the NF-*κ*B, Janus kinase-signal transducers and activators of transcription (STAT), AP-1, and nuclear factor of activated T cell pathways [66, 67]. As early as 1998, Ricote* et al*. [68] demonstrated that activation of PPAR*γ* results in reduced transcriptional activity of cytokine-induced inflammation-associated factors, such as* Ap-1*,* Nfkb*, and* Stat *genes in mouse myeloid progenitor cells. Additionally, PPAR*γ* reportedly plays an important role in improving insulin sensitivity [69], with clinical studies showing improvements in metabolic disorders and reduced inflammatory responses in type 2 diabetes patients treated with the PPAR*γ* agonist RG within 6 months after receiving coronary artery intervention [70]. In the present study, CGA treatment promoted preadipocyte differentiation by activating PPAP*γ*2 in a similar manner to RG treatment, although RG treatment promoted a greater increase in lipid accumulation relative to that observed following CGA treatment. These results implied that CGA prevented lipid accumulation, even in the presence of activated PPAP*γ*2, suggesting its potential efficacy as a PPAR*γ* agonist for clinical application.

## 5. Conclusions

This represents the first validation that CGA constitutes a PPAP*γ*2 agonist capable of effectively stimulating 3T3-L1 preadipocyte differentiation. However, our findings showed that CGA acted differently from RG in the area of fat metabolism during adipocyte differentiation and that TAG content was significantly higher in the RG-treated group relative to that in the CGA-treated group. This might be due to CGA enhancing lipolysis but not lipid synthesis, resulting in decreased lipid accumulation prior to preadipocyte differentiation. These data indicated that CGA represents a novel PPAR*γ*2 agonist different from RG; however, further study of the regulation mechanism associated with CGA-mediated activity on lipid metabolism is necessary to provide insight to its potential application for preventing insulin resistance and hyperglycemia.

## Figures and Tables

**Table 1:** Primer sequences used for real-time qPCR.

Gene	Primer sequence (5′–3′)
*Pparg2*	F:TCAAGGGTGCCAGTTTCG R:GGAGGCCAGCATCGTGT
*Pref1*	F:TCTCACGCACACTCACATCA R:CAACCTGGGGTCTCTCTCTG
*Cebpb*	F:GTTTCGGGACTTGATGCAAT R:AACCCCGCAGGAACATCT
*Srebp1*	F:AACCAGAAGCTCAAGCAGGA R:TCATGCCCTCCATAGACACA
*Rxra*	F:CCCAGCTCACCAAATGACCCT R:CTCGTTCCAGCCTGCCCGTA
*Plin*	F:TAGAGTTCCTCCTGCCACCA R:GTGCTGACCCTCCTCACAAG
*Dgat1*	F:TCCAGACAACCTGACCTACCGA R:CTCAAGAACTCGTCGTAGCAG
*Hsl*	F:AATCCCACGAGCCCTACCTCA R:CCTGCAAGGCATATCCGCTCT
*Actb*	F: CTTCTTTGCAGCTCCTTCG R: TTCTGACCCATTCCCACC

## Data Availability

All the data are available from Professor Zheng Wang (zhengw@hunau.edu.cn) upon reasonable request.

## References

[1] Nathan D. M. (2015). Diabetes: advances in diagnosis and treatment. *The Journal of the American Medical Association*.

[2] Ye J. (2011). Challenges in drug discovery for thiazolidinedione substitute. *Acta Pharmaceutica Sinica B (APSB)*.

[3] Ogurtsova K., da Rocha Fernandes J., Huang Y. (2017). IDF Diabetes Atlas: Global estimates for the prevalence of diabetes for 2015 and 2040. *Diabetes Research and Clinical Practice*.

[4] Song X., Jia H., Jiang Y. (2015). Anti-atherosclerotic effects of the glucagon-like peptide-1 (GLP-1) based therapies in patients with type 2 Diabetes Mellitus: A meta-analysis. *Scientific Reports*.

[5] Chatterjee S., Khunti K., Davies M. J. (2017). Type 2 diabetes. *The Lancet*.

[6] Nissen S. E., Wolski K. (2007). Effect of rosiglitazone on the risk of myocardial infarction and death from cardiovascular causes. *The New England Journal of Medicine*.

[7] Wei W., Wang X., Yang M., Smith L. C., Dechow P. C. (2010). PGC1beta mediates PPARgamma activation of osteoclastogenesis and rosiglitazone-induced bone loss. *Cell Metabolism*.

[8] Orchard T. J. (2006). The effect of rosiglitazone on overweight subjects with type 1 diabetes. *Diabetes Care*.

[9] Zhang K.-H., Huang Q., Dai X.-P. (2010). Effects of the peroxisome proliferator activated receptor-*γ* coactivator-1*α* (PGC-1*α*) Thr394Thr and Gly482Ser polymorphisms on rosiglitazone response in Chinese patients with type 2 diabetes mellitus. *Clinical Pharmacology and Therapeutics*.

[10] Lu Y., Ma D., Xu W., Shao S., Yu X. (2015). Effect and cardiovascular safety of adding rosiglitazone to insulin therapy in type 2 diabetes: a meta-analysis. *Journal of Diabetes Investigation*.

[11] Schreiber I., Dörpholz G., Ott C.-E. (2017). BMPs as new insulin sensitizers: Enhanced glucose uptake in mature 3T3-L1 adipocytes via PPAR*γ* and GLUT4 upregulation. *Scientific Reports*.

[12] Toyota Y., Nomura S., Makishima M., Hashimoto Y., Ishikawa M. (2017). Structure-activity relationships of rosiglitazone for peroxisome proliferator-activated receptor gamma transrepression. *Bioorganic & Medicinal Chemistry Letters*.

[13] Clifford M. N. (2000). Miscellaneous phenols in foods and beverages - Nature, occurrence and dietary burden. *Journal of the Science of Food and Agriculture*.

[14] Adedapo A. A., Adeoye B. O., Sofidiya M. O., Oyagbemi A. A. (2015). Antioxidant, antinociceptive and anti-inflammatory properties of the aqueous and ethanolic leaf extracts of Andrographis paniculata in some laboratory animals. *Journal of Basic and Clinical Physiology and Pharmacology*.

[15] Ahuja S., Kohli S., Krishnan S., Dogra D., Sharma D., Rani V. (2011). Curcumin: A potential therapeutic polyphenol, prevents noradrenaline- induced hypertrophy in rat cardiac myocytes. *Journal of Pharmacy and Pharmacology*.

[16] Tajik N., Tajik M., Mack I., Enck P. (2017). The potential effects of chlorogenic acid, the main phenolic components in coffee, on health: a comprehensive review of the literature. *European Journal of Nutrition*.

[17] Liu S.-L., Peng B.-J., Zhong Y.-L., Liu Y.-L., Song Z., Wang Z. (2015). Effect of 5-caffeoylquinic acid on the NF-*κ*B signaling pathway, peroxisome proliferator-activated receptor gamma 2, and macrophage infiltration in high-fat diet-fed Sprague-Dawley rat adipose tissue. *Food & Function*.

[18] Jiang R., Hodgson J. M., Mas E., Croft K. D., Ward N. C. (2016). Chlorogenic acid improves ex vivo vessel function and protects endothelial cells against HOCl-induced oxidative damage, via increased production of nitric oxide and induction of Hmox-1. *The Journal of Nutritional Biochemistry*.

[19] Deka S. J., Gorai S., Manna D., Trivedi V. (2017). Evidence of PKC binding and translocation to explain the anticancer mechanism of chlorogenic acid in breast cancer cells. *Current Molecular Medicine*.

[20] Zhang Z., Wang D., Qiao S. (2017). Metabolic and microbial signatures in rat hepatocellular carcinoma treated with caffeic acid and chlorogenic acid. *Scientific Reports*.

[21] Xue N., Zhou Q., Ji M. (2017). Chlorogenic acid inhibits glioblastoma growth through repolarizating macrophage from M2 to M1 phenotype. *Scientific Reports*.

[22] Sanchez M. B., Miranda-Perez E., Verjan J. C. G., de los Angeles Fortis Barrera M., Perez-Ramos J., Alarcon-Aguilar F. J. (2017). Potential of the chlorogenic acid as multitarget agent: Insulin-secretagogue and PPAR *α*/*γ* dual agonist. *Biomedicine & Pharmacotherapy*.

[23] Hakkou Z., Maciuk A., Leblais V. (2017). Antihypertensive and vasodilator effects of methanolic extract of Inula viscosa: Biological evaluation and POM analysis of cynarin, chlorogenic acid as potential hypertensive. *Biomedicine & Pharmacotherapy*.

[24] Palócz O., Pászti-Gere E., Gálfi P., Farkas O. (2016). Chlorogenic acid combined with lactobacillus plantarum 2142 reduced LPS-induced intestinal inflammation and oxidative stress in IPEC-J2 cells. *PLoS ONE*.

[25] Ghadieh H. E., Smiley Z. N., Kopfman M. W., Najjar M. G., Hake M. J., Najjar S. M. (2015). Chlorogenic acid/chromium supplement rescues diet-induced insulin resistance and obesity in mice. *Journal of Nutrition and Metabolism*.

[26] Martínez G., Regente M., Jacobi S., Del Rio M., Pinedo M., de la Canal L. (2017). Chlorogenic acid is a fungicide active against phytopathogenic fungi. *Pesticide Biochemistry and Physiology*.

[27] Wang L., Bi C., Cai H. (2015). The therapeutic effect of chlorogenic acid against Staphylococcus aureus infection through sortase A inhibition. *Frontiers in Microbiology*.

[28] Wu D., Bao C., Li L. (2015). Chlorogenic acid protects against cholestatic liver injury in rats. *Journal of Pharmacological Sciences*.

[29] Ye H., Jin J., Jin L., Chen Y., Zhou Z., Li Z. (2017). Chlorogenic Acid Attenuates Lipopolysaccharide-Induced Acute Kidney Injury by Inhibiting TLR4/NF-*κ*B Signal Pathway. *Inflammation*.

[30] Mikami Y., Yamazawa T. (2015). Chlorogenic acid, a polyphenol in coffee, protects neurons against glutamate neurotoxicity. *Life Sciences*.

[31] Park J. J., Hwang S. J., Park J.-H., Lee H.-J. (2015). Chlorogenic acid inhibits hypoxia-induced angiogenesis via down-regulation of the HIF-1*α*/AKT pathway. *Cellular Oncology*.

[32] Zheng S. Q., Huang X. B., Xing T. K., Ding A. J., Wu G. S., Luo H. R. (2017). Chlorogenic acid extends the lifespan of caenorhabditis elegans via insulin/IGF-1 signaling pathway. *Journal of Gerontology Series A Biological Sciences and Medical Sciences*.

[33] Cho A.-S., Jeon S.-M., Kim M.-J. (2010). Chlorogenic acid exhibits anti-obesity property and improves lipid metabolism in high-fat diet-induced-obese mice. *Food and Chemical Toxicology*.

[34] Jang B.-C. (2016). Artesunate inhibits adipogeneis in 3T3-L1 preadipocytes by reducing the expression and/or phosphorylation levels of C/EBP-*α*, PPAR-*γ*, FAS, perilipin A, and STAT-3. *Biochemical and Biophysical Research Communications*.

[35] Ruiz R., Jideonwo V., Ahn M. (2014). Sterol regulatory element-binding protein-1 (SREBP-1) is required to regulate glycogen synthesis and gluconeogenic gene expression in mouse liver. *The Journal of Biological Chemistry*.

[36] Tyagi S., Gupta P., Saini A. S., Kaushal C., Sharma S. (2011). The peroxisome proliferator-activated receptor: a family of nuclear receptors role in various diseases. *Journal of Advanced Pharmaceutical Technology & Research*.

[37] Lago R. M., Singh P. P., Nesto R. W. (2007). Congestive heart failure and cardiovascular death in patients with prediabetes and type 2 diabetes given thiazolidinediones: a meta-analysis of randomised clinical trials. *The Lancet*.

[38] Agrawal R., Jain P., Dikshit S. N. (2012). Balaglitazone: a second generation peroxisome proliferator-activated receptor (PPAR) gamma (*γ*) agonist. *Mini-Reviews in Medicinal Chemistry*.

[39] Zheng G., Qiu Y., Zhang Q.-F., Li D. (2014). Chlorogenic acid and caffeine in combination inhibit fat accumulation by regulating hepatic lipid metabolism-related enzymes in mice. *British Journal of Nutrition*.

[40] De Souza Marinho Do Nascimento D., Oliveira R. M., Camara R. B. G. (2017). Baccharis trimera (Less.) DC exhibits an anti-adipogenic effect by inhibiting the expression of proteins involved in adipocyte differentiation. *Molecules*.

[41] Hu E., Tontonoz P., Spiegelman B. M. (1995). Transdifferentiation of myoblasts by the adipogenic transcription factors PPAR*γ* and C/EBP*α*. *Proceedings of the National Acadamy of Sciences of the United States of America*.

[42] Lee H., Kim J., Park J. Y., Kang K. S., Park J. H., Hwang G. S. (2017). Processed panax ginseng, sun ginseng, inhibits the differentiation and proliferation of 3T3-L1 preadipocytes and fat accumulation in caenorhabditis elegans. *Journal of Ginseng Research*.

[43] Fujimoto T., Ohsaki Y., Cheng J., Suzuki M., Shinohara Y. (2008). Lipid droplets: a classic organelle with new outfits. *Histochemistry and Cell Biology*.

[44] Huang K., Liang X.-C., Zhong Y.-L., He W.-Y., Wang Z. (2015). 5-Caffeoylquinic acid decreases diet-induced obesity in rats by modulating PPAR*α* and LXR*α* transcription. *Journal of the Science of Food and Agriculture*.

[45] Liew C. W., Boucher J., Cheong J. K. (2013). Ablation of TRIP-Br2, a regulator of fat lipolysis, thermogenesis and oxidative metabolism, prevents diet-induced obesity and insulin resistance. *Nature Medicine*.

[46] Smas C. M., Sul H. S. (1993). Pref-1, a protein containing EGF-like repeats, inhibits adipocyte differentiation. *Cell*.

[47] Hudak C. S., Sul H. S. (2013). Pref-1, a gatekeeper of adipogenesis. *Frontiers in Endocrinology*.

[48] Sul H. S., Smas C., Mei B., Zhou L. (2000). Function of pref-1 as an inhibitor of adipocyte differentiation. *International Journal of Obesity*.

[49] Jang J., Jung Y., Chae S. (2018). Gangjihwan, a polyherbal composition, inhibits fat accumulation through the modulation of lipogenic transcription factors SREBP1C, PPAR*γ* and C/EBP*α*. *Journal of Ethnopharmacology*.

[50] Rosen E. D., Hsu C. H., Wang X. (2002). C/EBPalpha induces adipogenesis through PPARgamma: a unified pathway. *Genes & Development*.

[51] Herrera R., Ro H. S., Robinson G. S., Xanthopoulos K. G., Spiegelman B. M. (1989). A direct role for C/EBP and the AP-I-binding site in gene expression linked to adipocyte differentiation. *Molecular and Cellular Biology*.

[52] Christy R. J., Kaestner K. H., Geiman D. E., Daniel Lane M. (1991). CCAAT/enhancer binding protein gene promoter: Binding of nuclear factors during differentiation of 3T3-L1 preadipocytes. *Proceedings of the National Acadamy of Sciences of the United States of America*.

[53] Hwang C.-S., Mandrup S., MacDougald O. A., Geiman D. E., Lane M. D. (1996). Transcriptional activation of the mouse obese (ob) gene by CCAAT/enhancer binding protein *α*. *Proceedings of the National Acadamy of Sciences of the United States of America*.

[54] Sprott K. M., Chumley M. J., Hanson J. M., Dobrowsky R. T. (2002). Decreased activity and enhanced nuclear export of CCAAT-enhancer-binding protein *β* during inhibition of adipogenesis by ceramide. *Biochemical Journal*.

[55] Wu Z., Xie Y., Bucher N. L., Farmer S. R. (1995). Conditional ectopic expression of C/EBP beta in NIH-3T3 cells induces PPAR gamma and stimulates adipogenesis. *Genes & Development*.

[56] Oberfield J. L., Collins J. L., Holmes C. P. (1999). A peroxisome proliferator-activated receptor gamma ligand inhibits adipocyte differentiation. *Proceedings of the National Acadamy of Sciences of the United States of America*.

[57] Mueller E., Drori S., Aiyer A. (2002). Genetic analysis of adipogenesis through peroxisome proliferator-activated receptor *γ* isoforms. *The Journal of Biological Chemistry*.

[58] Ren D., Collingwood T. N., Rebar E. J., Wolffe A. P., Camp H. S. (2002). PPARgamma knockdown by engineered transcription factors: exogenous PPARgamma2 but not PPARgamma1 reactivates adipogenesis. *Genes & Development*.

[59] Rashid A. M., Lu K., Yip Y. M., Zhang D. (2016). Averrhoa carambola L. peel extract suppresses adipocyte differentiation in 3T3-L1 cells. *Food & Function*.

[60] Cheong L. Y., Suk S., Thimmegowda N. R. (2015). Hirsutenone directly targets PI3K and ERK to inhibit adipogenesis in 3T3-L1 preadipocytes. *Journal of Cellular Biochemistry*.

[61] Kowalska K., Olejnik A., Rychlik J., Grajek W. (2014). Cranberries (Oxycoccus quadripetalus) inhibit adipogenesis and lipogenesis in 3T3-L1 cells. *Food Chemistry*.

[62] Den Besten G., Bleeker A., Gerding A. (2015). Short-chain fatty acids protect against high-fat diet-induced obesity via a PPARgamma-dependent switch from lipogenesis to fat oxidation. *Diabetes*.

[63] Konishi Y., Kobayashi S. (2004). Transepithelial transport of chlorogenic acid, caffeic acid, and their colonic metabolites in intestinal caco-2 cell monolayers. *Journal of Agricultural and Food Chemistry*.

[64] Aoyagi R., Funakoshi-Tago M., Fujiwara Y., Tamura H. (2014). Coffee inhibits adipocyte differentiation via inactivation of PPAR*γ*. *Biological & Pharmaceutical Bulletin*.

[65] Du W.-Y., Chang C., Zhang Y. (2013). High-dose chlorogenic acid induces inflammation reactions and oxidative stress injury in rats without implication of mast cell degranulation. *Journal of Ethnopharmacology*.

[66] Clark R. B., Bishop-Bailey D., Estrada-Hernandez T., Hla T., Puddington L., Padula S. J. (2000). The nuclear receptor PPAR*γ* and immunoregulation: PPAR*γ* mediates inhibition of helper T cell responses. *The Journal of Immunology*.

[67] Lee H., Shi W., Tontonoz P. (2000). Role for peroxisome proliferator-activated receptor *α* in oxidized phospholipid-induced synthesis of monocyte chemotactic protein-1 interleukin-8 by endothelial cells. *Circulation Research*.

[68] Ricote M., Li A. C., Willson T. M., Kelly C. J., Glass C. K. (1998). The peroxisome proliferator-activated receptor-gamma is a negative regulator of macrophage activation. *Nature*.

[69] Li Z.-Y., Song J., Zheng S.-L. (2015). Adipocyte Metrnl Antagonizes Insulin Resistance Through PPAR*γ* Signaling. *Diabetes*.

[70] Wang G., Wei J., Guan Y., Jin N., Mao J., Wang X. (2005). Peroxisome proliferator-activated receptor-*γ* agonist rosiglitazone reduces clinical inflammatory responses in type 2 diabetes with coronary artery disease after coronary angioplasty. *Metabolism - Clinical and Experimental*.

